# Manual of Cattle-Feeding

**Published:** 1881-04

**Authors:** 


### REVIEWS.

---

MANUAL OF CATTLE-FEEDING. By HENRY P. ARMSBY, Ph.D. New York: John Wiley & Son, 1880. 8 vo, pp. 525.

It surely indicates a growing interest in veterinary science in our country that a book of the character which we find this to be should be published. The author has treated his subject in a clear and systematic manner, and he shows a thorough acquaintance with the scientific principles that underlie it. The book is divided into three parts. The first treats of the general laws of animal nutrition. There are chapters upon the composition of the animal body, upon the components of food, upon digestion and resorption, and upon various other topics connected with the digestion of food and its transformations in the body. Especial attention is given to the physiology of the formation of flesh and of fat. The principles upon which a flesh-giving diet and a fat-giving diet should be based are discussed in full. The second part treats of the different kinds of foods. Each one is taken in order, its analysis given, and its comparative digestibility shown. The third part is devoted to the feeding of farm animals. Here we have given, first, feeding standards; then the proper food for oxen, sheep, etc. The variations in this food, in accordance with the amount of work done or the amount of flesh or milk desired, are laid down. A chapter is also devoted to the feeding of growing animals, and another to the calculation of rations.

It will be seen that the subject of cattle-feeding is gone over very completely, and is treated in something more than a merely popular manner. The chemistry of food, the physiology of its digestion, its changes, and the laws which govern the production of flesh, of fat, of heat and of work, are stated briefly, but still with sufficient fulness and clearness. It is a work which can be understood by the man who has not had a medical education. but it is also one which ranks higher than a merely popular feeding manual.

The book meets a decided want in this country, and although a good deal of it is not original, but taken from German works, nevertheless, great credit is due to Dr. Armsby for the careful manner in which he has performed his part.

